# Cerium Oxide–Polysulfone Composite Separator for an Advanced Alkaline Electrolyzer

**DOI:** 10.3390/polym12122821

**Published:** 2020-11-27

**Authors:** Jung Won Lee, ChangSoo Lee, Jae Hun Lee, Sang-Kyung Kim, Hyun-Seok Cho, MinJoong Kim, Won Chul Cho, Jong Hoon Joo, Chang-Hee Kim

**Affiliations:** 1 Hydrogen Research Department, Korea Institute of Energy Research (KIER), 152 Gajeong-ro, Yuseong-gu, Daejeon 34129, Korea; leejw792@kier.re.kr (J.W.L.); cs.lee@kier.re.kr (C.L.); jhlee@kier.re.kr (J.H.L.); ksk@kier.re.kr (S.-K.K.); hscho@kier.re.kr (H.-S.C.); mj.kim@kier.re.kr (M.K.); chk14@kier.re.kr (C.-H.K.); 2 Department of Urban, Energy, and Environmental Engineering, Chungbuk National University, 1 Chungdae-ro, Seowon-gu, Cheongju, Chungbuk 28644, Korea; 3 Advanced Energy and System Engineering, Korea University of Science and Technology (UST), 217 Gajeong-ro, Yuesong-gu, Daejeon 34113, Korea; 4 Department of Advanced Material Engineering, Chungbuk National University, 1 Chungdae-ro, Seowon-gu, Cheongju, Chungbuk 28644, Korea

**Keywords:** alkaline water electrolyzer, ceria nanoparticle, diaphragm membrane, Zirfon separator, electrolytic cell

## Abstract

The intermittent and volatile nature of renewable energy sources threatens the stable operation of power grids, necessitating dynamically operated energy storage. Power-to-gas technology is a promising method for managing electricity variations on a large gigawatt (GW) scale. The electrolyzer is a key component that can convert excess electricity into hydrogen with high flexibility. Recently, organic/inorganic composite separators have been widely used as diaphragm membranes; however, they are prone to increase ohmic resistance and gas crossover, which inhibit electrolyzer efficiency. Here, we show that the ceria nanoparticle and polysulfone composite separator exhibits a low area resistance of 0.16 Ω cm^2^ and a hydrogen permeability of 1.2 × 10^–12^ mol cm^–1^ s^–1^ bar^–1^ in 30 wt% potassium hydroxide (KOH) electrolyte, which outperformed the commercial separator, the Zirfon PERL separator. The cell using a 100 nm ceria nanoparticle/polysulfone separator and advanced catalysts has a remarkable capability of 1.84 V at 800 mA cm^−2^ at 30 wt% and 80 °C. The decrease in the average pore size of 77 nm and high wettability (contact angle 75°) contributed to the reduced ohmic resistance and low gas crossover. These results demonstrate that the use of ceria nanoparticle-based separators can achieve high performance compared to commercial zirconia-based separators.

## 1. Introduction

The power-to-gas (PtG) process converts electric power into chemical energy to stably store energy for a long time [1,2,3]. The electrical energy is consumed for water splitting to produce hydrogen and oxygen using an electrolyzer. Polymer electrolyte membrane (PEM) electrolysis has a quick start, high flexibility, and better coupling properties with dynamic and intermittent systems [4,5]. Water electrolysis with alkaline electrolytes is recognized as a mature and reliable system. It is a technology based on cost and simplicity compared to other types of electrolysis [6,7,8,9]. Recently, alkaline electrolysis employing anion exchange membranes (AEMs) demonstrated a high rate of over 2 A cm^−2^ [10,11,12] and high flexibility that are comparable to PEM electrolysis [13,14]. However, AEMs still suffer from polymeric degradation and contamination from CO_2_ in the air after a performance test [15,16,17]. Thus, most commercial alkaline electrolyzers are operated while equipped with a diaphragm or porous separator as a membrane while circulating the electrolyte solution [18].

Composite porous separators are typically composed of hydrophilic inorganic materials combined with polymers. Polyantomonic acid was first developed for use in alkaline water electrolyzers because of its high alkali-resistance [19]. Potassium titanate [20], titania [21], sintered nickel plate [22], and zirconia [23,24,25] showed excellent stability in electrochemical systems as separator materials. Cerium oxide also shows excellent alkali-resistance and is utilized in many fields, such as magnetics, phosphors, alloys, and catalysis [26]. CeO_2_ was used to direct ethanol PEM fuel cells as a Pd support material because of the lowering of the onset energy of ethanol [27].

The pore structure of the separator, including pore size distribution, porosity, and average pore diameter, is a key performance parameter that influence the conductivity, permeability, and bubble point pressure of the porous separator [28,29]. The pore structure can be adjusted by controlling the nanoparticle, ratio of polymer in the suspension, mixing temperature, evaporation, etc. However, very little attention has been paid to the effect of nanoparticle size on the porous structure of the separator [30,31].

In this study, we synthesized a ceria-polysulfone (PSU) composite separator for use in an alkaline electrolyzer via a phase inversion process and performed in comparison with the commercial Zirfon PERL separator and the composite separators. The effect of the particle size of CeO_2_ on the key performance criteria was conducted regarding bubble point pressure, ionic resistance, and H_2_ permeability. The ceria-based separator was tested with respect to cell performance to evaluate the practicability in alkaline water electrolyzers.

## 2. Experimental

### 2.1. Materials

N-methyl-2-pyrrolidione (NMP, ≥99%) and polyvinylpyrrolidone (PVP, K90 represents average molecular weights of 360,000) were supplied by Sigma-Aldrich (St. Louis, MO, USA), while polysulfone (PSU, Udel) was acquired from Solvay (Beveren, Belgium). CeO_2_ nanoparticles were purchased from US Nano Research (Houston, TX, USA) with various sizes of 10 to 30, 50, and 100 nm. Polyphenylene sulfide mesh (PPS, PPS80PW, PVF Mesh and Screen Technology) was used as support. Distilled water (Pure Power I, Human science) and a 45 wt% potassium hydroxide (KOH) solution (DAEJUNG) were used for preparing 30 wt% KOH solution. The commercial Zirfon PERL separator was purchased from Agfa (Mortsel, Belgium).

The physical properties of the purchased CeO_2_ nanoparticles are summarized in Table 1. The C20 separator was composite with CeO_2_ nanoparticles with sizes of 10 nm and 30 nm; the Brunauer–Emmett–Teller (BET) surface area and average pore diameter were 64 m^2^ g^−1^ and 9 nm, respectively. The CeO_2_ nanoparticles of 50 nm and 100 nm had similar BET surface areas (22–24 m^2^ g^−1^) and average pore diameters (16 to 17 nm). 

### 2.2. Separator Preparation

The CeO_2_/PSU composite separators were synthesized by film casting method. PVP and PSU were blended with NMP using the mixing device (RED150-D, Pendraulik, Springe, Germany) and CeO_2_ nanopowder was added into the homogenous slurry. The composite ratio of CeO_2_ to PSU was fixed at 80/20 wt%. Afterwards, the suspension was cast using a doctor blade apparatus. The PPS mesh was inserted in the center of the separator. After casting, the separator was dried in a convection oven (JEIO TECH. Co., Ltd., Daejeon, Republic of Korea) at a temperature of 90 °C for 20 min. The solidified sample was deposited in distilled water to extract the solvent at 5 °C. The specimen (thickness ~460 μm) was washed with deionized water several times and stored in deionized water. The synthesized separators were named the C*X* separator (where *X* is the average size of the CeO_2_ nanopowder). For example, the C50 separator indicates a separator that was prepared with 50 nm CeO_2_ nanoparticles.

### 2.3. Electrode Preparation

Nickel foam material (NI003852) with porosity 110 pore per inch was obtained from Goodfellow Corp (Xiamen, China). In order to remove the oxides and impurities on the surfaces, Ni foams were soaked at 20 wt% NaOH and 80 °C for 5 min and immersed in 18 wt% HCl and 25 °C for 5 min. The Raney type nickel cathodes were prepared by a physical vapor deposition (PVD). The aluminum was sputtered on to porous nickel foams (~1.6 mm thickness) by direct current magnetron sputtering (DC 300 W), then heat-treated at 610 °C for 150 min. After the heat-treatment, the aluminum-coated nickel foam was immersed overnight in 30 wt% potassium hydroxide and a temperature of 80 °C for selective aluminum leaching in gamma phase nickel-aluminum alloys. The NiFe layered double hydroxide (LDH) anode was fabricated by a pH-controlled in-situ growth method. A clean iron foam was immersed into nickel sulphate solution and the pH level was controlled by continuous oxygen sparging into the solution for 7 h at a temperature of 50 °C. The external voltage was not applied during the preparation process. After 7 h, the sample was taken out and rinsed with deionized water and ethanol. The prepared LDH anode exhibited a Ni: Fe ratio of 1:1 containing SO_3_^2−^ as intercalated anions and the α-FeOOH/NiFe LDH structure has been constructed successfully on the iron substrate.

### 2.4. Characterization

The scanning electron microscopy (SEM, S-4800, HITACHI, Tokyo, Japan) was used to investigate the microstructures of the prepared separators. To confirm construction of microscopic particles in the prepared separators, elemental mapping was conducted by Energy Dispersive X-ray Spectroscopy (EDS, Unti^®^ Max, Oxford Instruments, Abingdon, UK). Prior to analysis, osmium was coated on the specimens to enhance the conductivities. The physical properties of fabricated separators, such as the porosity, average pore diameter, pore volume, and surface area, were studied by a gas adsorption (Tristar 3000, Micromeritics, Norcross, GA, USA) and a mercury porosimeter (Auto Pore 9520, Micromeritics, Norcross, GA, USA). The X-ray diffraction (XRD, Rigaku/D/max-2000 Ultima, Tokyo, Japan) analysis was conducted to determine the crystalline structure of composite separators and the Zirfon separator.

The bubble point pressure corresponding to the gas tightness was measured using the American Society for Testing Materials (ASTM) F316 procedure. The separator was located inside of the cell and was contacted with water and air on the upper side and the lower side, respectively.

To measure the conductivities of the CeO_2_/PSU composite separators, electrochemical impedance spectroscopy (SP240, BioLogic Science Instruments, Paris, France) with through-plane H-type cell (VB8-S, EC Frontier, Kyoto, Japan) was used. The specimen was placed between the cells with an active area of 3.14 cm^2^. The measurement was conducted at 30 wt% KOH with an AC amplitude corresponding to 10% of the applied current density in the frequency range of 10 kHz to 0.1 Hz, and the conductivities of separators were obtained at 2 kHz.

To confirm the wettability of separators, the contact angle was measured by Washburn’s approach. The separator samples were prepared a width (~20 mm) and a length (~50 mm) and dried in an oven for 12 h at a temperature of 90 °C. The resulting separator was deposited into a liquid (30 wt% KOH). Regarding time, the mass change was observed and the contact angle was recorded using the Washburn equation in detail elsewhere. [32].

A separator was prepared to an area of 24.45 cm^2^ and inserted into the center of the cell to measure the hydrogen permeability. The electrolyte (30 wt% KOH solution) was used to thoroughly fill both sides of the cell at a temperature of 30 °C. The differential pressure was controlled from 1.1 to 1.5 bar through a flowmeter (5850E, Brooks, Hatfield, PA, USA) and a back pressure controller (44-2361-24, Tescom Corp., Elk River, MN, USA). The higher-pressure side and lower side are referred to as the cathode and anode, respectively. The mass change permeated though the separator was obtained. The hydrogen permeability $\varepsilon_{H_{2}}^{Darcy}$ (mol cm^−1^ s^−1^ bar^−1^) driven by the differential pressure can be defined as:

(1) $$\varepsilon_{H_{2}}^{Darcy}=\frac{K}{\eta }S_{H_{2}}P_{H_{2}}^{cat}$$

where *K* denotes the electrolyte permeability (cm^2^), $S_{H_{2}}$ is the solubility of hydrogen in the electrolyte (mol m^–3^ bar^–1^), $P_{H_{2}}^{cat}$ is the partial hydrogen pressure at the cathode side (bar), and the viscosity of the electrolyte is represented by η (bar s). According to Darcy’s law, the molar permeation flux density ($\Phi_{H_{2}}^{Darcy}$ in mol cm^–1^ s^–2^) of hydrogen via the separator driven by the differential pressure can be calculated as follows:

(2) $$\Phi_{H_{2}}^{Darcy}=-\varepsilon_{H_{2}}^{Darcy}\frac{\Delta p}{d}$$

where Δp denotes the differential pressure (bar) of both sides of the cell and d indicates the thickness of the separator.

### 2.5. Electrolysis Tests

Rectangle-shaped electrodes and the separators were prepared based on 34.56 cm^2^ as an active area and assembled in a zero-gap type electrolytic cell with nickel-based porous transport layers (PTLs), bipolar plates (BPs), and a current collector. The cells were operated using 30 wt% KOH electrolytes circulated with a flow rate of 450 sccm at a temperature of 80 °C. Cell voltage was recorded at a current density in a range from 0 to 2000 mA cm^–2^ by a potentiostat equipped with a booster (Advanced Power system N7970A, Agilent Technologies, Santa Clara, CA, USA). Galvanostatic electrochemical impedance spectroscopy (GEIS) was obtained at a current density of 200 mA cm^−2^ in a frequency range from 10 kHz to 1 Hz.

## 3. Results and Discussion

**Figure 1:**
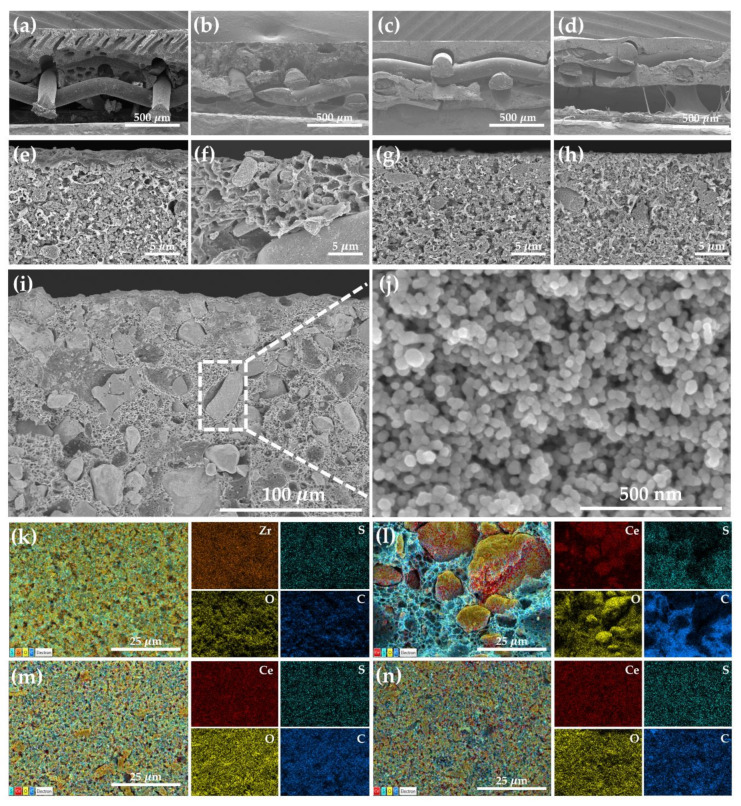
Cross-sectional SEM images, low-magnification (**a**) Zirfon, (**b**) C20 separator, (**c**) C50 separator, (**d**) C 100 separator and higher magnification (**e**) Zirfon, (**f**) C20 separator, (**g**) C50 separator, (**h**) C100 separator, and (**i**) aggregates of C20 separator (**j**) inset of agglomerated CeO_2_ nanoparticles. SEM-EDS elemental mapping analyses of (**k**) Zirfon, (**l**) C20 separator, (**m**) C50 separator, and (**n**) C100 separator.

The cross-sectional SEM images of the CeO_2_-based separators and Zirfon separators are shown in Figure 1. From the low-magnification images (Figure 1a–d), the Zirfon separator has visible pores across the separators (Figure 1a). The C20 separator also had large pores (Figure 1b). On the contrary, no visible pores were observed for the C50 and C100 separators (Figure 1c,d). The polymeric layer and agglomerates were observed in the high-magnification images (Figure 1e,f). The Zirfon separator showed a layer of polymer several micrometers thick (Figure 1e). On the contrary, a distinct layer was not visible for the CeO_2_-based separators (Figure 1g,h). The composites of ZrO_2_ and PSU were randomly distributed for the Zirfon separator (Figure 1e) and the CeO_2_-based separators, including the C50 and C100 separators (Figure 1g,h). However, a few micrometers of agglomerations were clearly observed within the bulk of the C20 separator (Figure 1f). The CeO_2_ nanoparticles agglomerated to form large aggregates, as shown in (Figure 1i,j). CeO_2_ nanoparticles and polysulfone were well mixed with perfect compatibility for the C50 and C100 separators. SEM-EDS elemental mapping analysis showed that CeO_2_ nanoparticles were aggregated in the C20 separators. (Figure 1l). Meanwhile, they were well dispersed in the C50 and C100 separators (Figure 1m,n).

**Figure 2:**
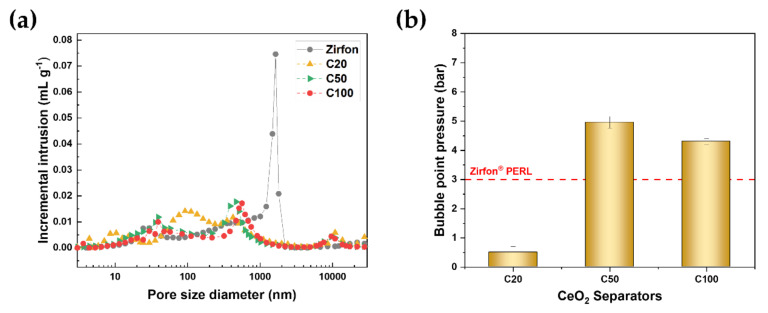
(**a**) Hg incremental intrusion volume curves and (**b**) bubble point pressure of the composite separators and Zirfon separator.

The mercury-pore size distributions of the composite separators and the Zirfon separator are shown in Figure 2a. The separators showed a bimodal pore distribution. Smaller pores ranging from 10 to 50 nm are correlated to the pores between CeO_2_ nanoparticles, and the second larger pores are associated with the interconnected pores of the polymer matrix [25]. There was a narrow peak with a strong intensity in the micro-sized pores in the Zirfon separator. Meanwhile, the CeO_2_-based separators exhibited a decrease in the micro-size pores with reduced intensity. In addition, these separators showed a broad pore distribution from 10 to 300 nm with a relatively higher intensity than the Zirfon separator. The Hg porosimetry results of the separators are summarized in Table 2. The Zirfon separator had the largest average pore size of 116 nm, which is related to a high portion of large pores. The C20 separator had the smallest average pore size of 51 nm, which increased as the size of CeO_2_ nanoparticles increased. The CeO_2_-based separators exhibited a first broad pore distribution in the range of 10 to 50 nm, which is associated with the shift in peaks shifted to the left toward smaller pore diameters.

The bubble point pressure (BPP) for the composite separators is plotted in Figure 2b. Bubble point pressure is the minimum pressure required to force the liquid out of the pores, which is related to the pore size of the micropores of the membrane. According to Laplace’s equation, the bubble point pressure depends on the maximum pore size (R_max_) [31]. The C50 and C100 separators had larger BPP than the Zirfon separator, which exhibited the largest average pore diameter (~116 nm). The BPP decreased from 5 ± 0.2 to 4 ± 0.1 bar as CeO_2_ nanoparticle size increased. A high BPP is inversely related to the small average pore size, which is in line with the average pore size in Table 2.

**Figure 3:**
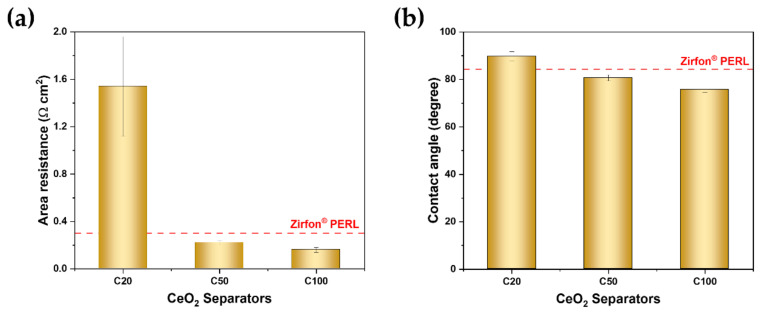
(**a**) Area resistance and (**b**) wettability of the composite separators and Zirfon.

The area resistance and wettability as a function of synthesized CeO_2_ nanoparticle size are shown in Figure 3. The classical alkaline electrolyzer uses 25 to 30 wt% KOH solution as an electrolyte because the polymeric membrane is still in research stages due to its instability under alkaline condition [33]. The porous separator should be filled with a concentrated electrolyte solution. The conductivity and wettability of the porous separator are responsible for the ohmic resistance.

The area resistance of the Zirfon separator, which was prepared with 40 nm ZrO_2_ nanoparticles, was approximately 0.3 Ω cm^2^. The area resistance of the C20 separator was 1.5 ± 0.5 Ω cm^2^, and it decreased to 0.16 ± 0.02 Ω cm^2^ as the CeO_2_ nanoparticle size increased (Figure 3a). The wettability decreased from 90 to 75° as the synthesized CeO_2_ nanoparticle size increased (Figure 3b). The area resistance and wettability decreased as the porosity increased, as shown in Table 2 and Figure 3b, respectively. These results indicated that the preparation of the separator with larger CeO_2_ nanoparticles was recommended due to an increase in the average pore diameter of the prepared separator while maintaining high BPP.

The porous separator provides ionic conductivity by allowing aqueous electrolytes to pass through it while separating the evolving gases [28]. Aqueous electrolytes can permeate through the separator driven by the differential pressure. This permeability increased as porosity increased. The separator with high porosity showed low ohmic resistance but high permeability. Thus, there is a trade-off between ionic resistance and H_2_ permeability. The Zirfon separator has a micropore with a strong intensity (Figure 2), through which dissolved hydrogen in the electrolyte could permeate driven by the differential pressure [24], leading to a limited part-load range of the classic alkaline electrolyzer. Meanwhile, the polymeric electrolyte membrane in the PEM electrolyzer typically has nanopores of 2 to 5 nm, which is responsible for low gas crossover by the differential pressure. A porous separator with smaller pores should be developed to reduce gas crossover driven by the differential pressure.

**Figure 4:**
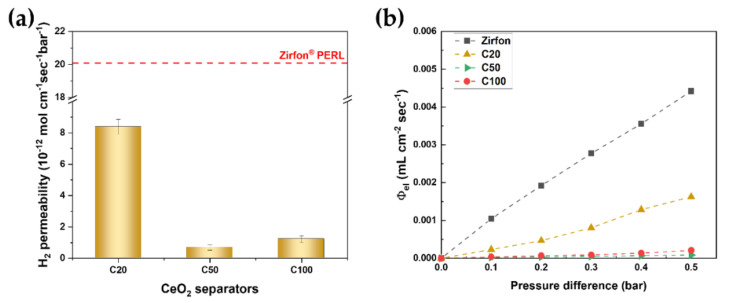
(**a**) H_2_ permeability (**b**) volumetric permeation flux density of the composite separators and Zirfon.

The H_2_ permeability of the Zirfon separator was as high as 20 × 10^−12^ mol cm^−1^ s^−1^ bar^−1^, as shown in Figure 4a. On the contrary, the H_2_ permeability of the C20 and C50 separators was 8.38 ± 0.48 × 10^−12^ mol cm^−1^ s^−1^ bar^−1^ and 0.69 ± 0.18 × 10^−12^ cm^−1^ s^−1^ bar^−1^, respectively. Figure 4b shows the volumetric permeation flux density of the electrolyte via separators as a function of the absolute differential pressure. The cell temperature was 30 °C. The Zirfon separator showed the highest volumetric permeation flux density, followed by the C20, C100, and C50 separators. These results are consistent with the average pore sizes of the separators in Table 2. Notably, the C100 separator exhibited a low ohmic resistance (0.16 Ω cm^2^) and low hydrogen permeation flux density (1.2 × 10^–12^ mol cm^−1^ s^−1^ bar^−1^), which comprise only about 50% of the Zirfon separator’s resistance and 6% of the Zirfon separator’s H_2_ permeability, respectively.

**Figure 5:**
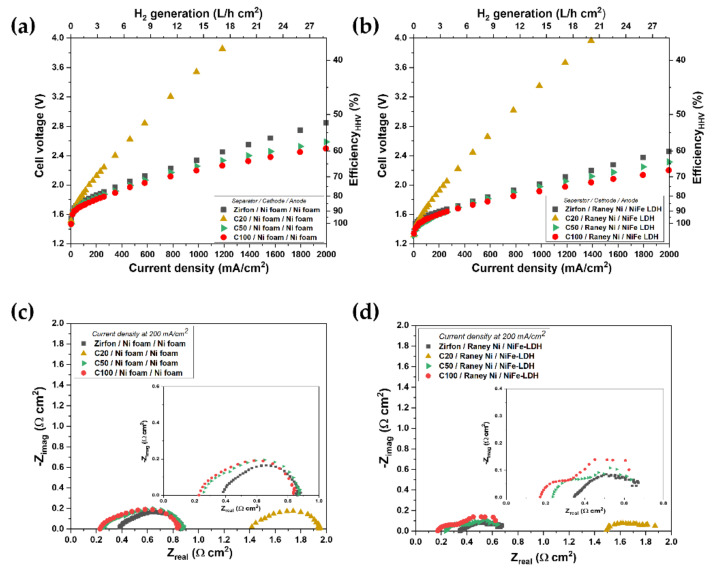
In-situ polarization curve and impedance spectra of a single cell with (**a**,**c**) Ni foam/cathode—Ni foam/anode and (**b**,**d**) Raney Ni/cathode—NiFe Layard double hydroxide (LDH)/anode in 30 wt% potassium hydroxide (KOH) solution at 80 °C (the inset showed the closed-up at low impedance range).

The polarization characteristics of the cell in various combinations with catalysts and separators are shown in Figure 5. The cells were equipped with a nickel-foam electrode at a temperature of 80 °C and 30 wt% KOH solution and are presented in Figure 5a. The differences between the separators increased with the higher current density owing to the ohmic resistance. The C20 separator showed poor performance, while the C100 separator showed the best performance. The cell equipped with the C100 separator achieved 59% voltage efficiency at 2000 mA cm^−2^. The current–voltage polarization curves for Raney-Ni as the hydrogen evolution reaction (HER) electrode and LDH as the oxygen evolution reaction (OER) electrode are shown in Figure 5b. The cell employing the C100 separator showed a higher performance of 2.2 V at 2000 mA cm^−2^ than that of the Zirfon separator (2.5 V at 2000 mA cm^−2^), enhancing the voltage efficiency by 67%. The ohmic resistances of the separators measured by the Nyquist plot (Figure 5c,d) were in line with the ex situ results (Figure 3). The results suggest that the CeO_2_-based separator could contribute to improving the voltage efficiency due to high wettability while lowering the H_2_ permeability resulting from the reduced average pore size.

**Figure 6:**
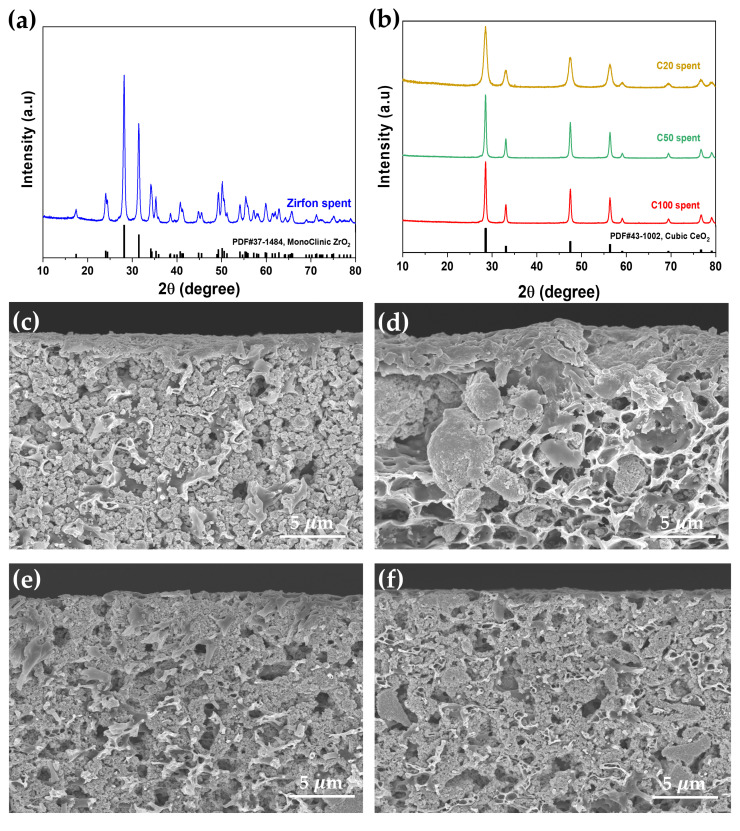
XRD patterns of spent (**a**) Zirfon separator, (**b**) CeO_2_-based separators, including C20, C50 and C100, and cross-section SEM images of spent (**c**) Zirfon separator, (**d**) C20 separator, (**e**) C50 separator and (**f**) C100 separator. The “spent” represent the separator after electrochemical test up to 2000 mA cm^-2^ at 30 wt% KOH and 80 °C.

The structural stability of the spent Zirfon and CeO_2_-based separators after the in-situ cell test was further studied by XRD and cross-sectional SEM analysis as given in Figure 6. The XRD pattern of the Zirfon separator shows no peak shift compared with the standard card of monoclinic ZrO_2_ (Figure 6a) and the CeO_2_-based separators exhibit good agreement with standard JCPDS Cubic CeO_2_ phase without any other impure peaks (Figure 6b). Their cross-sectional SEM images are shown from Figure 6c–f. The morphology of spent Zirfon and CeO_2_-based separators show the negligible difference as compared to pristine separators (Figure 1e–h). These results suggest that the CeO_2_/PSU composites exhibit their chemical and physical stability under harsh operating conditions, such as high current density (~2000 mA cm^−2^), temperature of 80 °C, and 30 wt% KOH electrolyte.

## 4. Conclusions

In this paper, we presented a CeO_2_-based porous separator that outperforms the commercial Zirfon separator in terms of ohmic resistance and H_2_ permeability. The CeO_2_-based porous separator was synthesized by varying the CeO_2_ nanoparticle size. The porous separator with the addition of 100 nm CeO_2_ nanoparticles reduced the average pore size while enhancing the wettability. The C100 separator-equipped alkaline cell achieved 800 mA cm^−2^ at 1.84 V for the Raney-Ni-catalyzed HER and NiFe LDH-catalyzed in 30 wt% KOH electrolytes and it exhibited much low H_2_ permeability. The results demonstrated in this study show that CeO_2_ nanoparticles can be used as organic materials for highly ionic conductive and low gas permeable porous separators.

## Figures and Tables

**Table 1:** The physical properties for CeO_2_ nanoparticles.

Sample	BET Surface Area (m^2^ g^−1^) ^a^	Pore Volume (cm^3^ g^−1^) ^a^	Average Pore Diameter (nm) ^a^
C20	64	0.154	9
C50	23	0.094	16
C100	24	0.104	17

^a^ Brunauer–Emmett–Teller (BET) method (Tristar 3000, Micromeritics).

**Table 2:** Physical properties of composite separators and Zirfon.

	Unit	C20	C50	C100	Zirfon
Total pore area	[m^2^ g^−1^]	40.1 ± 0.5	27.8 ± 0.13	18.6 ± 0.3	17.4 ± 0.53
Average pore diameter	[nm]	51 ± 5	62 ± 3	77 ± 3	116 ± 10
Bulk density	[g mL^−1^]	1.05 ± 0.03	1.21 ± 0.025	1.26 ± 0.013	1.08 ± 0.29
Apparent density	[g mL^−1^]	2.31 ± 0.35	2.56 ± 0.27	2.32 ± 0.3	2.37 ± 0.5
Porosity	[%]	54 ± 3	53 ± 2	46 ± 2	54 ± 5
Thickness	[μm]	455 ± 20	465 ± 30	460 ± 25	500 ± 50
Bubble point pressure	[bar]	0.5 ± 0.2	5 ± 0.2	4 ± 0.1	3 ± 0.5
